# Collective actions in crisis

**DOI:** 10.3389/fsoc.2026.1723300

**Published:** 2026-03-10

**Authors:** Hatem H. Alsaqqa

**Affiliations:** 1 Public Health Department and Deanship of Scientific Research, Al-Quds University, Jerusalem, Palestine; 2 Palestinian Ministry of Health, Gaza, Palestine

**Keywords:** collective action, crisis, decision-making, sensemaking, technology

## Abstract

When a crisis strikes, people want quick and accurate information, and fast responses can assist to fill in the gaps left by the crisis. Crises present enormous hurdles to population decision-making. Creating excellent judgments is also difficult since it entails making sacrifices for the greater good. This perspective study shows that social conventions and norms are ways for resolving collective action concerns. When group cohesiveness is the norm, it becomes easier to address difficulties related to collective action. However, the crisis compels us to reevaluate our beliefs and return to our fundamental values. One of the best of these values at a larger level is collective action. As the crisis leads to shortage in time, resources and choices which limits the individual preferences or narrows the discrepancies, it puts the collective action as a priority. The study indicates that collaborative stakeholders must be able to communicate effectively with one another in order to make informed decisions in difficult situations and to engage, manage, and recover from a crisis. The researcher considers what is learned about crisis, decision-making and making sense in tumultuous situations, and argues for principles that underpins these concepts. The researcher investigates when and how shared meanings enable more useful or adaptive behavior. People's ability to react to crises is also at an inflection moment, particular thanks to technological advancements and social media. This study provides the crisis mechanism as well as a review of enabling technologies and their potential in the collective action.

## 1 Introduction

Collective action is defined as actions made by two or more individuals with the goal of achieving the same collective good (Marwell and Oliver, 1993). Individuals or engaged communities must have clearly defined internal roles and be properly structured in a leadership structure in order to fulfill their collective goals. Moreover, the creation of social movements, as well as voting and bidding habits, have all been studied using collective action approaches (Bimber et al., 2005).

According to Ostrom's research, a community is more inclined to work together when its members would be similarly damaged by passivity and benefited by action. The existence of a socially troubled concerns becomes a major explanation for more demanded collective collaboration (Singleton and Taylor, 1992).

On the contrary, crises arise when interests are at risk, the consequences of actual acts are unclear, and a prompt response is necessary (Coleman, 2004). High levels of stress impair decision-making by increasing the likelihood of failure, stiffening decision-making, and lowering tolerance for complexity (Holsti, 1978). Collective thinking, which is typified by an overestimation of the largely closed group decision-making process and expects uniformity, is encouraged in the crisis response environment (Stubbart, 1987).

Collective action entails a contradiction between individual and group reason in this sense. Despite the fact that everyone is tempted not to participate, low levels of contribution make the entire community worse. Individuals are prevented from engaging in collective action contexts by one or both of two factors: the urge to free-ride where the desire to free-ride implies a willingness to delegate responsibility for collective action to others (Singleton and Taylor, 1992), and the worry of lack of effectiveness.

Most crises may necessitate a combination of resources, including money, equipment, sanctions, regulations, standards, locations, summits, experts, and insurance. However, finding and evaluating solutions to the “collective action dilemma” is a major focus of collective action theory and research. The literature on collective action identifies three sorts of solutions: • Coordination: act in a coordinated manner to resolve the situation. • Work together on a common goal: develop a cooperative instrument (or a fund) to address the situation. • Burden sharing: distributing the costs to reduce the effects (Blondin and Boin, 2020).

The formation of norms and social conventions that enable collaboration can be facilitated by repeated interaction. The logic goes that repeated engagement leads to the formation of interconnected relationships, shared norms, and trust (Putnam, 1993). Interactions over time may even lead to the formation of a shared identity among agents, which would drastically alter their choices and hence speed up cooperation (Ekengren, 2018).

Nonetheless, according to crisis communication academics, policymakers are logical individuals who follow a chronological analytical path and choose among competing options to select the optimal crisis response approach for the scenario. It's also challenging to cope with ambiguity, make decisions without information, find that reaction networks were not well prepared to effectively communicate with an audience that is concerned.

The establishment of policy window frames (Kingdon and Stano, 1984) resulting from the creation of appropriate problems and policy suggestions stimulated by, or justified in the name of crisis experience, and the increased perceptions of potential influence generating community demands of action, are two examples of the legacy of crisis (Stern, 1997).

## 2 Mechanisms of decisions and collective action in crisis situations

From the literature mentioned above, a crisis is clarified as an unexpected, sudden, and unclear incident that poses a serious threat and leaves only a limited amount of time to make a decision (James and Wooten, 2010; Pearson and Clair, 1998). Industrial crises [such as Chernobyl or the Challenger tragedy; see (Weick, 1988)], and natural disasters are examples of low-probability, high-impact events [such as the Mann Gulch fire (Weick, 1993)].

However, crises bring with them a rush of rapidly evolving massive corpus of essential knowledge from a variety of sources. In such cases, a frequent technique is to wait until all relevant information is accessible, then consider it thoroughly before making a well-informed conclusion. Making managerial decisions during these crucial times through group activities offers the potential advantage of enabling each group member to contribute special knowledge to the problem, such as epidemiological, clinical, or economic data (Sandler, 2015).

The researcher claims that crises provoke a slew of unique problems, preventing teams from making and implementing appropriate decisions. Three obstacles stand out in terms of collective action: efficient deliberation, deciding for the public purpose and sticking to choices. When judgments have such a clear conflict between individual and collective consequences (Weber et al., 2004), deciding on one's or group's advantage (i.e., collaborating) needs self-control (Sheldon and Fishbach, 2011; Martinsson et al., 2012). Crises disturb these patterns because they drastically alter our collaborative style.

Therefore, it is critical to take care from the start to comprehend the issue and to make use of the diversity and alterations of viewpoints that surround it, in order to ensure that all of the issue's critical parts are given the proper care and attention (Al-Dabbagh, 2020). From the perspective of information processing, such collective actions may have an advantage over individual actions because (1) members can learn from each other and (2) they can incorporate all available data to make a different decision than each ordinary person alone (Schulz-Hardt and Mojzisch, 2012). Individual members do not have enough information to make the best option in these cases; hence they are regularly alluded to as concealed profiles (Stasser et al., 1989).

Nonetheless, challenges in times of crisis necessitate even more dedication. Wise decisions are typically difficult to make since they require prioritizing the collective good over immediate self-interest (Van Lange et al., 2013). To make better decisions in concealed profile situations (under time constraints), teams must usually take their time to gather and integrate relevant data. For example, arguing for various decision options boosted team information processing, at least in terms of boosting the intensity of conversation (Greitemeyer et al., 2006). In the end, what is expected of decision-making, is that people accept the conclusion and respond appropriately to it, despite a high level of skepticism around all provided choices and under enormous psychological strain as a total collapse process (Al-Dabbagh, 2020).

Eventually, because escalation impairs the accuracy of crisis decision-making, (Paraskevas 2006) underlined the importance of detection systems and feedback mechanisms for detecting the expansion of the crisis and associated events. Hence, in light of the developing problem, decision-making must be regenerated to change the living system. From describing the problem to gathering and classifying information that determines the status of options, the process of making decisions involves various phases and then creates a better choice in a way that enhances decision-makers' efficiency (Al-Dabbagh, 2020).

## 3 Sensemaking in crisis and how it happens

Sensemaking raises the issue of whether shared meanings are beneficial rather than hazardous in a crisis. Weick offers some insight into this problem, claiming that “knowledge” can help address the apparently disparate roles that sensemaking can play in mitigating crises (Maitlis and Sonenshein, 2010).

Although it is acknowledged that sensemaking is a social construction process, it is simple to become engrossed in theorizing and then overlook the significance of the social processes that facilitate sensemaking and the shared beliefs that may result from them (Maitlis, 2005; Weick, 1995).

Indeed, researchers hotly discuss the extent to which sensemaking interpretations must be communicated to enable collaborative action (Balogun and Johnson, 2004). Some researchers even argue that the level to which diverse meanings permit the same behavioral outcomes, is more important than the level to which common meaning exists (Donnellon et al., 1986).

Groups that focus on two essential processes—updating and doubting—are less likely to fall victim to several problems that turn seemingly identical meanings into important blinders [e.g., (Nickerson, 1998)]. Three key themes that must be considered while making sense:

**Commitment:** which is defined as the devotion and engagement people have in upholding social connections and carrying out their social obligations, is the cornerstone for producing sense. This is due to the fact that people frequently create excuses after the fact to justify their behavior (Weick, 1999).

**Identity:** which is defined as the collection of characteristics that define an individual or a group, such as ideas, personality traits, style, and/or behaviors, is a concept that researchers more broadly believe is fundamental for sensemaking. It is another important sort of shared meaning that frequently appears in sensemaking studies about crises and change [see especially (Weick, 1999)]. When challenges arise in such situations, the value of identity becomes even more apparent.

**Expectations:** which is defined as implicit guidelines that direct behavior and ideas in an approach that is socially acceptable, is a sort of shared meaning that can be evaluated and combined with evidence to form meanings, as Weick (Weick, 1999) points out. Individuals then compare subsequent clues to this meaning, progressively gaining trust in a situation identification.

Nevertheless, public investigations have been described as longer-term reactions to crises that can lead to learning (Brown, 2000). They show a lot about how shared sense is formed and delivered during a crisis when micro-level sensemaking practices produce the macro social order (Gephart, 1993; Gephart et al., 1990).

## 4 Mindfulness, communication, and technology in crisis

Complex processes like technology network dynamics present interesting empirical questions concerning communicative relationships. It emphasizes how members perform personal interactions and varied activities by viewing groupings of collaboration and involvement (Bimber et al., 2012). To understand the significance of collective mindfulness, it is crucial to understand that awareness is more than just “how finite attention is distributed” (March, 1994).

When it comes to mindfulness, the quality of awareness is just as much about maintaining focus. Many researchers see technology as less of a conclusion and more of a manageable choice if it is adopted by deliberate, ongoing collective awareness practiced by intelligent, devout, trustworthy, and self-respecting individuals (Campbell, 1990). It was recognized that engaged communal mindfulness is complicated and unusual combination of human attentiveness, skill, reverence, communication, negotiation, paradoxical action, boldness, and prudence.

However, communication promotes a feeling of shared purpose among actors who play a variety of characters. It is a way of informing global community stakeholders and the general public in diverse countries about the potential hazard, the need for evidence-based mitigation initiatives, and a collaborative approach. When public officials are confronted with potential dangers, decision-making becomes an organized activity with four unique components: (1) cognition, (2) communication, (3) coordination, and (4) control (Fligstein and McAdam, 2021).

Cognition is he “capacity to determine the degree of rising danger to which a community is subjected and to act on that information” is a key component of crisis management (Comfort, 2007). Importantly, cognition provides the transition to action. It constitutes not simply the perception of risk to self but also comprehension of the risk. Crucially, the shift from thought to action is provided by cognition. It includes understanding the risk to others in addition to the risk to oneself (Ansell et al., 2010). The basic element of empathy in cognition fosters a human bond with those who are at risk and motivates collective actions for the well of the community general population. The public struggled to understand the scope, severity, and deadly nature of the crisis.

Communication problems or limits are likely to result in sub-optimal crisis management, especially if there is no consistent relations entity that can operate as a center for information collection and distribution (Comfort, 2007). Furthermore, because it is very possible for users to provide, and it is simple to contribute private resources to important services, the arrival of modern facilities is lessening essential engagement efforts and incentives (Bimber et al., 2005). Choosing between competing systems and applications based on needs and utility is one of the initial challenges. In this regard, contemporary developments have the potential to increase properly implemented communication and assist in lessening the distressing consequences of dangerous circumstances (Ansell et al., 2010).

However, technology-based communal action, also known as contemporary collective action, is the product of technological innovation. As present communications are more complex and can readily occur through the internet, the extensive application of technology threatens the limitations of classic collective action theory (Vogt et al., 2011).

Social media, by acting as a catalyst for immersion, has become widely adopted, shared, practiced, and deeply rooted in societies. In addition, collective action theory is used to further explain the creation of social media by taking into account the pursuing of common goals and collective connections (Flanagin et al., 2006). Only the special mechanism by which micro-level activity progresses to the macro-level and produces global, representative behavior can be said to be an emergent system (Tim et al., 2013).

Ultimately, this process represents a prosocial phenomenon in which sustained and beneficial collective actions drive the customization of social media, enabling it to serve as a catalyst for integration and the creation of an affiliated community. That contemporary technology can circumvent the traditional constraints of collective action; specifically, it encourages large-group activity to enhance the visibility of new media, as connections and activities are now influenced by established norms (Lupia and Sin, 2003). If risk assessment and collaborative techniques can be effectively linked to the phenomenon, social media may be used as an effective crisis intervention platform to meet a variety of demands that arise during various stages of catastrophes (Kaewkitipong et al., 2012).

## 5 Managing crisis and the coordination challenge

During a crisis, there is a strong consensus among scholars that some types of coordination, collaboration or cooperation are required (e.g., Kapucu et al., 2010). These terms are used in this literature to describe related but separate types of collective action that enforce certain reward systems on individual players, causing them to either align with or break from a collectively rational response. Decentralized techniques emphasize dynamic interactions across actors in response to crises, whereas centralized approaches focus on building a coordinated crisis-management strategy (e.g., Waugh and Streib, 2006).

However, it is impossible to totally control all hazards, human flaws, and unexpected situations. Crisis management, on the other hand, is characterized as a collection of planning and reactive activities aimed at containing the risk and its implications. Almost every crisis response has an operating scheme as well as a strategic component. Organizing and supporting a coordinated response to an urgent threat is known as strategic crisis management.

The fundamental concept of crisis management involves the social network's capacity to effectively carry out the following activities, which will help produce the most effective actions to mitigate the impact of crises: • Sense-making: governing the mechanism by which strategic crisis managers come to a common understanding of the unfolding threat and its ramifications. This necessitates the gathering, analysis, and sharing of knowledge about the risk and its implications as it develops. • Critical decision-making: making effective and acceptable strategic judgments (while avoiding operational ones) in the near and long term. • Coordination: enabling the execution of planned activities and strategic decisions by encouraging participants in networks' response to collaborate and carry out their responsibilities. • Meaning-making: explaining what is occurring, whatever is being done to correct the situation and mitigate the severity, and providing concrete guidance for moving forward to all concerned parties (Ansell and Bartenberger, 2016; Zollo and Winter, 2002).

It is difficult to make key decisions and manage a complicated network response to effectively interact with anxious residents and involved stakeholders. Yet, a rational approach to crisis may be acceptable when situations are relatively straightforward and unchanging, but what works in these circumstances may not work in times of “unruly difficulties” (Ansell and Bartenberger, 2016).

Thus, crisis managers with less experience are prone to request additional information in the face of uncertainty. They are taught to consider the short- and long-term ramifications of critical actions, especially by creating and evaluating several scenarios. They often try to manage anxiety with facts when designing communications for a worried audience. In fact, crisis managers make do with what they have, draw conclusions based on a few basic concepts, stutter forward depending on their people's expertise and give communications that delicately balance imagery and facts (Kingdon and Stano, 1984).

## 6 Patterns of collective activities and social capital

Patterns of actions are learned and established as a sequence of collective activities, through which the population systematically produces and adapts its operating routines in search of enhanced effectiveness (Zollo and Winter, 2002). As with other coordinated operations, information transmission is essential to incorporating a new project or collective idea into team effectiveness. If subunits fail to communicate information and integrate improvisation, they may perceive the situation differently (Roux-Dufort and Vidaillet, 2003). Different groups or subsystems may have different criteria or ethical codes, which in addition to political difficulties, may impede the ability to respond quickly in a crisis (Beamish, 2002). Boundary bridging allows information to be translated from one group to another in order to ensure active engagement.

However, experts' interaction and criticism, particularly in crisis situations, help to direct continuous improvisation (Faraj and Xiao, 2006). On the other hand, social norms are shared conceptual frameworks about what acts are required, permissible or banned (Ostrom, 2005). Because these conventions are first learned through family connections, they may differ from one culture to another (Bowles and Gintis, 2002). These norms are built and dismantled over time and in different contexts as a result of human contact (Crawford and Ostrom, 1995). Thus, face-to-face communication is a factor that significantly promotes respect and as a result, collective action in a group (Ostrom, 2000).

Moreover, the component of trust, as determined by individual interactions, is a critical factor in resolving collective action difficulties, since it determines relationships and levels of engagement (Ostrom, 2017). These bonds are based on social capital, which is defined as trust, concern for group members, willingness to follow community rules, and the ability to penalize those who do not (Ostrom, 2000; Ostrom and Ahn, 2009). Interpersonal norms, relationships and forms of civic involvement, as well as formal and informal organizations, are all characterized as social capital generators (Kremer et al., 2019). Therefore, sociologists should focus on the various practices that trigger collective action during times of crisis. Investigation of the process is notably important in demonstrating how people collaborate to confront the existential anxiety and uncertainty caused by crises.

Nonetheless, providing members with social sources of support, trust and caring in times of desperation, are irreplaceable for cultivating effective interactions (House et al., 1988). Perceiving members as part of a larger outraged group rather than as isolated individuals during a crisis might lessen and transform the paralyzing sentiments of dread and grief that are usually experienced into more invigorating feelings of indignation and faith (Heidemann, 2018).

Consequently, these individuals can become empowered agents, capable of generating and executing tangible sets of strategic practices. Position-taking practices, on the other hand, can evolve and crystallize into more structured and long-term forms of collective action and mobilization, which can then promote the long-term replication of supporting various identities and interests.

In such cases, the embryonic yet regular forms of stance-taking that arose in response to a crisis can start to draw from and evolve into bigger and more long-lasting social capital or social movements. Nonetheless, understanding the situational practices people employ to adopt solidarity stances during pivotal moments might assist in clarifying the processes that support the development of social movements during crises and breakdowns (Heidemann, 2018).

## 7 The significance of leadership and social networks

People celebrate their “true leaders” when crisis leadership reduces stress and stability returns. In a broader sense, crisis leadership has been defined to quickly adjust, assist their organizations, and make strategic choices in the face of tremendous uncertainty. In order to maintain survival and generate chances for long-term success, leadership during crises frequently requires restructuring organizational frameworks and strategies. However, leaders affect not only policies but also how individuals interact, receive information, and deal with emotional discomfort. As a result, they play a critical role in establishing a social climate that inspires people to act and gives them the confidence to adjust to crisis situations.

Therefore, there is a strong correlation between good leadership and citizen responses. A social identity approach to leadership is based on encouraging people to “act as if they are a group” (Weick and Roberts, 1993). Crisis leadership must therefore go beyond the specific management abilities and skills that individual leaders either have or lack and concentrate on a relational approach (Haslam, 2020). Moreover, citizens' confidence in the society, its officials, and the directives themselves is crucial for effective community compliance with such circumstances (Freimuth et al., 2014). Therefore, it is possible to view institutional trust as both a result and an indicator of efficient management and communication (Yang et al., 2015).

Nonetheless, all members of the community benefit from mobilization and collective action generated by identification as community members and the importance of social identity over personal identities (Tajfel and Turner, 2001). Relationships and social networks are vital because they promote a sense of identification and belonging (Hopkins et al., 2019), inspire group action, and are critical to community members' health. Several variables, such as information quality, accessibility, and coordination between many actors, affect how well social networks perform in crisis management. Recognizing the importance of social networks and incorporating them into plans for crisis preparedness, response, and recovery is crucial.

Moreover, it is indisputable that social networks contribute to the resilience of communities in the face of disasters. After a crisis, they help communities reconstruct their lives, coordinate activities, and exchange information. As an example and similar to natural disasters, the COVID-19 pandemic disrupts organizational and personal behaviors and necessitates societal resilience. The ability of a social system to proactively respond to and heal from disturbances that are seen by the system as being outside the typical and anticipated shock range is referred to as resilience in catastrophe literature (Alam et al., 2018). The key to resilience is increasing maneuver-level adaptability.

In order to deal with shocks and the ensuing instability, communities must develop, enhance, and/or use their resources in an evolving and interactive process known as community resilience (McCrea et al., 2014). During COVID-19, a number of nations experienced overwhelmed health systems, semi-closed economies, and a consequent shortage of protective gear and services. Resilience is expected to help societies adjust throughout this phase of the health crisis, meaning that they will continue to function as “naturally” as feasible. Other phases of community resilience can help communities get ready for a catastrophe, recover from one, or even adapt after one (Elmqvist et al., 2019).

## 8 Conclusion

When the activities of two or more individuals or actors are necessary to achieve a goal, collective action emerges. However, the crisis offers diverse opportunities to explore the fundamental question of organizing the public to respond appropriately during a crisis. Taking this approach to collective action advances knowledge and study about how and why actors make complicated decisions.

When the advantages of a policy change are considerable and concentrated among a group of actors, there is a strong incentive for the group to act together. Resolving conflicts between overlapping concerns might become more difficult as the number of actors with an interest in the outcome increases.

It seems that collective action in crisis can harmonize policy-makers agendas around broad message blocks that invite diverse involvement and coordinate this engagement through fine-grained media applications. Indeed, collective action is currently being made with the feedback from a variety of sources and fields in order to maximize the number of learned lessons and to obtain the best reasonable options. A common knowledge influence, motivated from collective action can contribute to a reluctance of information exchange.

This study suggests deeper understanding of crisis mechanisms in the management of contentious collective action. When considering the circumstances of effective leadership and coordination, this perspective study may assist in balancing viewpoints that have emphasized collective action framing, technology and decision-making.

## Data Availability

The original contributions presented in the study are included in the article/supplementary material, further inquiries can be directed to the corresponding author.
